# Seroprevalence of HIV, HBV and HCV in Persons Referred to Hamadan Behavioral Counseling Center, West of Iran

**Published:** 2011-01-01

**Authors:** F Keramat, P Eini, M M Majzoobi

**Affiliations:** 1 Department of Infectious Diseases, Hamadan University of Medical Sciences, Hamadan, Iran

**Keywords:** HIV, HBV, HCV, Injection drug user, Prison, Iran

## Abstract

**Background:**

Human immunodeficiency virus (HIV), hepatitis B virus (HBV) and hepatitis C virus (HCV) are three important prevalent infections all over the world. The aim of this study was to determine seroprevalence of HIV, HBV and HCV infections and high risk behaviors in persons who referred to the behavioral counseling center of Hamadan, west of Iran.

**Methods:**

This was a cross-sectional study which was done on 379 persons who referred to the behavioral counseling center of Hamadan. All persons after obtaining the informed consent were tested for serologic markers including HBs Ag, HCV-Ab and HIV-Ab by ELISA and western blot methods.

**Results:**

Of the 379 persons, 71.5 % (271 cases) were male and 28.5% (108 cases) were female. HIV infection was reported in 4% (15) of persons. HBV and HCV infections were reported in 2.9% (11 cases) and 35.6% (135 cases), respectively. The most common high risk behaviors were injection drug user and history of prison with 52.5% (199 cases) and 40.4% (153 cases), respectively.

**Conclusion:**

According to the results, injection drug users and prisoners are at the highest risk for HCV, HIV and HBV infections.

## Introduction

Human immunodeficiency virus (HIV), hepatitis B virus (HBV) and hepatitis C virus (HCV) are three prevalent and important viral infections throughout the world. HIV infection is a sexual transmitted infection over the world, the most prevalent transmission rout of which, chiefly in developing countries, is Heterosexual.[1] At present, it is estimated that 37 million adults are living with HIV over the world, two-thirds of which are in sub Saharan Africa.[1]

On the basis of the last statistics until March 2010, 20975 HIV patients were recognized in Iran that 92.6% and 7.4% of whom were male and female, respectively. The peak of age distribution of HIV infections was 25-34 years, and on the basis of present statistics, injection drug user (IDU) is the most common transmission (69.8%) of HIV infection in Iran, and also 476 HIV patients was reported in Hamadan until March 2010 (Medical Education, Remedy and Health Ministry of Iran). Hepatitis B is one of the prevalent viral infections all over the world. It is estimated that 170 million infected people are living with it. In the endemic era, vertical transmission is the most rout of HBV spreading, but in lower prevalent era, majority of routs are via sex and blood transfusion. Also, prevalent rate of HBS Ag in blood donors varies between 5-10%.[2] Surveys show that risk of HBV transmission is 1/63000-500000 of transfused blood.[3][4] In western countries, HBV infection is higher in certain groups including IDUs, dialysis patients, persons who require repeated blood transfusion and homosexual men.[1][5] HCV also is a prevalent infection and is estimated that about 500 million are living with it throughout the world. Transmission routs of HCV are blood transfusion and other percutaneous ways including narcotic injection. The chance of sexual transmission has been estimated to be less than 5%.[2] Rate of transfusion-associated Hepatitis C is 1/1600000 infused blood. The most expressive HCV infection is sub clinical which can progress to chronic active hepatitis, cirrhosis and hepatic failure.[6]

The aim of this study was to determine the seroprevalence of HIV, HBV and HCV infections and associated risk factors in persons who referred to the behavioral counseling center of Hamadan in west of Iran.

## Materials and Methods

This was a cross-sectional study which was done on 379 referred persons to the Bbehavioral Counseling Center of Hamadan in west of Iran for evaluation of HIV, HBV and HCV serologic markers from March 2005 until January 2007. All data such as age, sex and after obtaining the informed consent, high risk behaviors of persons who referred to the center were recorded in the questionnaires after counseling by specialist of infectious disease. Permission required for these tests before performing the tests was acquired by a written permission from all individuals. Blood samples were tested by ELISA in Hamadan Blood Transfusion Center. For having a strong confirmation, all of HIV-Ab and HCV-Ab positive samples were confirmed by Western blot and positive tests were considered as hepatitis C or HIV infections. The Research Committee of Hamadan University approved this study. Statistical analysis was performed by using SPSS software for windows (ver. 15).

## Results

**Fig. 1:**
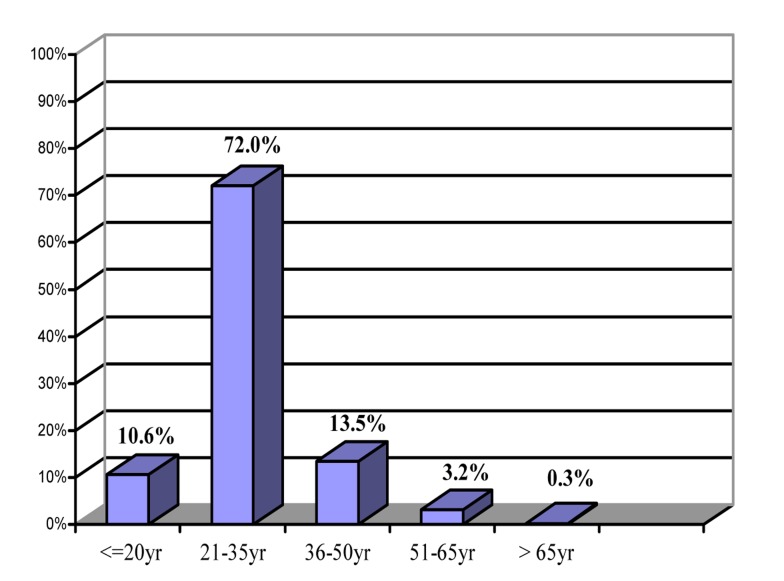
Age distribution of referred persons to the behavioral counseling center of Hamadan.

Of the 379 cases, 271 (71.5%) were male and 108 (28.5%) were female, with the mean age being 29.7±9.5 years in the range of 13-80 years (Figure 1). HIV infection was reported in 4% (15 cases), also 2.9% (11 cases) and 35.6% (135 cases) for HBV and HCV infections, respectively. The highest risk behaviors among these persons were injection drug use (52.5%) and then history of prison (40.4%) (Table 1). Co-infection of these three viral infections only was seen in a 33 years IDU man with history of prison. However, co-infection of two viral infections was seen in 4.5 % (17 cases) including 13 cases of HIV and HCV infections, 3 cases of HCV and HBV infections and 1 case with HIV and HBV infections.

**Table 1:** Distribution of high risk behaviors in referred persons to the behavioral counseling center of Hamadan

**High risk behaviors**	**N0. (%)**
Injection Drug Users (IDUs)	199 (52.5)
History of prison	153 (40.4)
History of tattoo	59 (15.6)
HBs-Ag positive patient in family	55 (14.5)
Wives of the IDUs patients	43 (11.3)
Unsafe sexual contact	29 (7.7)
HIV positive patient in family	27 (7.1)
HCV positive patient in family	12 (3.2)
History of dental procedures	5 (1.3)
Use of common blade	1 (0.3)
History of cupping	1 (0.3)

Distribution of HIV, HBVand HCV infections in these patients is showed in Table 2.

**Table 2:** Distribution of demographic characteristics in referred persons to the behavioral counseling center of Hamadan according to HIV, HCV and HBV infections

	**HIV (n=15)**	**HBV (n=11)**	**HCV (n=135)**
Sex			
Male	15 (100%)	9 (81.8%)	125 (92.6%)
Female	---	2 (18.2%)	10 (7.4%)
Age group			
≤ 20y	---	---	3 (2.2%)
21-35y	14 (93.3%)	9 (81.1%)	104 (77%)
36-50y	1 (6.7%)	1 (9.1%)	23 (17%)
51-65y	---	1 (9.1%)	5 (3.7%)
> 65y	---		

HCV infection was reported in 135 cases, while 126/135 cases were IDUs and 126/199 IDUs had HCV positive tests (63.3%). 125 (92.6%) of HCV positive were male and 10 cases (7.4%) were female. The most common age group of HCV patients (77%) was between 21-35 years old (Table 2).

## Discussion

HIV, HBV and HCV are three important and prevalent viral infections all over the world.[1]

In this study 71.5% of volunteers referred to the behavioral counseling center of Hamadan were male which is explainable with due attention to greater high risk and digression behaviors among men. Numerous incidences of HBV, HCV and HIV infections among homosexual and bisexual men in the different parts of USA confirm increases of acquiring these viral infections.[1][2][7][8][9][10]

In our study, 72% of referred persons were in the range of 21-35years. In developing countries, numerous factors including population growth (chiefly in adolescent and young adults), emigration from rural eras to towns, wars and poverty have caused serious damage through high risk behaviors. Peoples in the range of 21-35 years have much sexual activity, therefore incidence of sexual high risk behaviors is more prominent. Also with due attention to their problems, morality and behavioral diversity, addiction and criminality are greeter in young adults.[1] In present study, HIV and HBV infections were found in 4% (15 cases) and 2.9% of patients (11 cases), respectively.

In southern Iran, Davarpanah et al. (2008) determined the demographics and high-risk behaviors in HIV positive individuals in southern Iran during 2004-2005. The risk behaviors were 40.8% IDU, 16.4% unprotected sexual contact, 32.6% both IDU and unprotected sexual contact, 1.6% blood transfusion, 7.9% other high risk behaviors including tattooing, shared blade and knife injury and 0.7% had unknown high risk behavior. Maternal transmission was not observed.[11] In another study Davarpanah et al. (2007) investigated the seroprevalence of HBS Ag in patients with HIV infection in Shiraz, southern Iran showing that among 227 patients, 17 subjects were tested positive for HBS.[12] Davarpanah et al. (2009) also studied the hepatitis C viral genotypes in HCV monoinfected and human immunodeficiency virus (HIV)/HCV co-infected patients in southern Iran and demonstrated that among 273 HCV infected patients, HCV-RNA was detected in 238 subjects. 50 had HIV/HCV co-infection, among whom 88%, 92% and 56% had a positive history of intravenous drug use, being in prison, and tattooing, respectively. 188 subjects showed HCV monoinfection.[13]

Beech et al. reported in 150 homelessness adolescent that 16% were HIV positive and also revealed 17% HBV and 12% HCV positivity. Relationship between these results and high risk sexual behaviors, lack of HBV vaccine and non-existence of prevention programs are propounded in this study.[14] However, prevalence of HIV and HBV infections in our study was lower than Beech's study.

Hussain et al. reported the co-infections of HIV, HCV, HBV and syphilis in patients who had referred to STD clinics. Blood samples were obtained from 863 patients (635 female, 228 male). Results revealed 21 (2.4%) were infected by HIV, 25 (2.9%) HBV, 9 (1%) HCV and 47 (5.4%) syphilis.[15] The number of HBV infection is equal to our study. Co-infections of HIV-HBV in 2 cases (0.2%), HBV-HCV in 1 case (0.1%) and HIV-syphilis in 1 case (0.1%) were found but no case of triple viral infections of these four diseases were detected.[15] Therefore, in our research, coinfections were more common, including synchronization of HIV, HCV and HBV which was seen in only 1 case but two viral infections were existent in 17 cases (4.5%) including HIV-HCV (13), HCV-HBV (3 cases) and HIV-HBV (1 case). In the present study, 135 (35.6%) cases had HCV infection which consisted of a significant percentage. The most important routes of HCV transmission were substance injection and blood product transfusion and less important sexual contact.[2] In this study, 52.5% of volunteers referred to the behavioral counseling center and 126 (93.5%) of 135 HCV cases were IDU that confirms IDU is the most prevalent rout of HCV transmission or, in other words, hepatitis C virus is an epidemical event in addict populations of Iran. Also, the second referred groups to the behavioral counseling center were persons with history of prison (40.4%) 68% of whom were HCV infected.

Romia et al. compared three groups including high risk injecting drug users, blood products consumers, and patients undergoing chemotherapy with the control group regarding serologic markers of HIV, HBV and HCV. HCV positive serologic markers in each of three groups were more than the control group. Also, HBV infection in groups 2 and 3 unlike group 1 was more than the control group. No case of HIV was seen in three groups. This article recommends the need of screening tests in high risk groups.[16]

Bagheri et al. evaluated the prevalence of HBS-Ag in 104236 blood donors from 1980 to 1992 that 2.96% were positive and Male/Female ratio was equal to 1.1/1 and married/single as 1.2/1.[17] In another survey in Hamadan, incidence of HBV, HCV and HIV infections in 96 repeated blood recipients, were evaluated. HIV or HBV were not reported but the most important hepatitis in this region was HCV, because 49% of them had HCV-Ab in their serum samples.[18]

Kaur and Marshello screened 233 referred persons to STI clinics for HIV, HBV, HCV and syphilis, and their results revealed a rate of HIV-1 (3%), HBS-Ag (3%), HCV (0.8%) and syphilis 21%.[19] Also, Garg et al. evaluated 46957 blood donors for the same tests and results were apparent, incidence of HIV was 0.44%, HBV 3.44%, HCV 0.29% and VDRL 22%.[20]

Nanu et al. screened serologic markers in 132093 blood donors and exhibited frequency of HIV to be 0.55%, HBS-Ag 2.5%, HCV 1.49% and syphilis 0.52%.[21] However in our study, seroprevalence of HIV, HBV and HCV infections were higher than Nanu's study.

Patel screened 60780 dedicated bloods in Mumbai that 0.26%, 1.7% and 0.78% of whom were positive for HIV, HBV and HCV, respectively.[22] In the study of Gupta et al., in 44064 blood units in Lodhiana, HIV was reported 0.08%, HBS-Ag 0.6%, HBc-Ab 0.11%, HCV1 0.09% and syphilis 0.85%.[23]

Ruan et al. screened 379 IDUs showing that 71% of patients were HCV positive, 11.3% HIV positive and co-infections of the both were 11.3%.[24] However, in our research, 126 (93.3%) 0f 135 HCV positive were IDUs and 13 cases were HIV positive and coinfection of HIV-HCV was seen in 17 cases (4.5%).

Taketa et al. screened 98 IDUs, commercial sex workers and 50 men by STDs and reported the first manner of HCV infection via hematogenous contact (IDUs), so the first route of HIV transmission is similar to HCV, but second manner is sexual contacts, and the last as they reported HBV that was transmissible through sexual and hematogenous contacts.[25]

In conclusion, according to these results, it is defined that IDU and prison are the highest risk factor for acquisition of HBV, HCV and HIV infections. Screening of HCV, HBV and HIV infections in persons with high risk behaviors and establishing of behavioral counseling centers or STD clinics can help in early detection of them and reduce blood born (in IDUs) and sexual transmitted infections in high risk groups.
